# The MHC class II antigen presentation pathway in human monocytes differs by subset and is regulated by cytokines

**DOI:** 10.1371/journal.pone.0183594

**Published:** 2017-08-23

**Authors:** Justin Lee, Hanson Tam, Lital Adler, Alexandra Ilstad-Minnihan, Claudia Macaubas, Elizabeth D. Mellins

**Affiliations:** 1 Department of Pediatrics, Program in Immunology, Stanford University, Stanford, California, United States of America; 2 Department of Biology, Stanford University, Stanford, California, United States of America; "INSERM", FRANCE

## Abstract

Monocytes play a critical role in the innate and adaptive immune systems, performing phagocytosis, presenting antigen, and producing cytokines. They are a heterogeneous population that has been divided in humans into classical, intermediate, and non-classical subsets, but the roles of these subsets are incompletely understood. In this study, we investigated the expression patterns of MHC class II (MHCII) and associated molecules and find that the intermediate monocytes express the highest levels of the MHC molecules, HLA-DR (tested in n = 30 samples), HLA-DP (n = 30), and HLA-DQ (n = 10). HLA-DM (n = 30), which catalyzes the peptide exchange on the MHC molecules, is also expressed at the highest levels in intermediate monocytes. To measure HLA-DM function, we measured levels of MHCII-bound CLIP (class II invariant chain peptide, n = 23), which is exchanged for other peptides by HLA-DM. We calculated CLIP:MHCII ratios to normalize CLIP levels to MHCII levels, and found that intermediate monocytes have the lowest CLIP:MHCII ratio. We isolated the different monocyte subsets (in a total of 7 samples) and analyzed their responses to selected cytokines as model of monocyte activation: two M1-polarizing cytokines (IFNγ, GM-CSF), an M2-polarizing cytokine (IL-4) and IL-10. Classical monocytes exhibit the largest increases in class II pathway expression in response to stimulatory cytokines (IFNγ, GM-CSF, IL-4). All three subsets decrease HLA-DR levels after IL-10 exposure. Our findings argue that intermediate monocytes are the most efficient constitutive antigen presenting subset, that classical monocytes are recruited into an antigen presentation role during inflammatory responses and that IL-10 negatively regulates this function across all subsets.

## Introduction

Monocytes originate from hematopoietic stem cells in the bone marrow and comprise ~10% of blood leukocytes in humans. Activated monocytes operate as innate effectors during inflammatory and pathogenic responses, performing phagocytosis, producing cytokines and various other mediators [1, 2]. Monocytes also participate in adaptive immunity as antigen presenting cells [3]. Monocytes contribute to the pool of tissue macrophages in certain tissues (intestine, for example) and under certain inflammatory conditions, but recent work demonstrates that monocytes are not the precursor cells of most tissue macrophages during homeostasis or under some inflammatory conditions, with most tissues macrophages originating from embryonic precursors that colonize tissues prenatally [2, 4]. These discoveries are leading to a reassessment of monocyte function, both at homeostasis and during inflammatory responses, and the realization that monocytes and monocyte-derived cells may play important roles alongside macrophages and dendritic cells [2–4].

Monocytes are a heterogeneous population. In humans, two major subsets were initially identified based on the surface expression of CD14, the lipopolysaccharide (LPS) receptor, and CD16, the low affinity Fc receptor for IgG (FcγRIII). These subsets have been named classical monocytes (CD14++, CD16^-^), encompassing ~80–90% of the total monocytes and corresponding to the mouse Ly6C++ subset, and non-classical monocytes (CD14+, CD16++), ~10% of total monocytes and corresponding to the Ly6C+ in the mouse. A third subset, expressing both CD14 and CD16 and named intermediate subset (CD14++CD16+), was subsequently identified in humans, and constitutes ~10% of total monocytes [5]. It has also been suggested that the CD14 classical subset gives rise sequentially to the intermediate and to the non-classical subset [6].

The distinction between the 3 human monocyte subsets remains somewhat arbitrary, given the absence of markers that unambiguously distinguish them. However, phenotypic and gene expression studies suggest subset-specific functions [6–8]. It is also likely that further heterogeneity will be uncovered [9].

One differentiating feature revealed by transcriptional analyses is that intermediate monocytes express high levels of message from major histocompatility complex class II (MHCII) antigen processing and presentation genes [7, 8], compared to the classical and non-classical subsets. Constitutively expressed by professional antigen presenting cells (APCs), MHCII molecules, HLA-DR, -DQ, and -DP in humans, present peptides derived from endogenous or exogenous proteins. The biosynthetic pathway of MHCII molecules begins in the endoplasmic reticulum, where a dedicated chaperone, invariant chain (Ii), interacts with newly synthesized MHCII heterodimers, stabilizing them and preventing premature loading of ligands into the MHCII groove. MHCII/Ii complexes travel to endosomal compartments where Ii is digested by proteases, leaving a residual nested set of fragments called class II-associated Ii peptide, or CLIP, in the peptide-binding groove. In late endosomal compartments, CLIP is released and exchanged for other peptides. For most alleles, this exchange requires the action of HLA-DM, an MHCII homolog that also stabilizes empty MHCII and edits the peptide repertoire in favor of tight-binding peptides [10]. MHCII/peptide complexes interact with αβ T cell receptors on CD4+ T cells, to trigger adaptive immune responses.

The combined action of Ii and HLA-DM and other factors modulate the expression of MHCII at the cell surface. In this study, we analyzed expression of MHCII and MHCII related molecules in the 3 monocyte subsets. We assessed the ratio of surface MHCII/CLIP to total MHCII as a measure of HLA-DM function [11], both at baseline and following cytokine stimuli. These investigations shed light on mechanisms underlying diverse capabilities of the three human monocyte subsets.

## Materials and methods

### Sample collection and preparation

De-identified blood samples were obtained from healthy adult donors at the Stanford Blood Center; the work was conducted with approval from the Administrative Panels on Human Subjects Research from Stanford University. The blood was collected in heparin tubes (BD Vacutainer, BD, Franklin Lakes, NJ); peripheral blood mononuclear cells (PBMCs) were isolated by density gradient centrifugation using LSM Lymphocyte Separation Medium (MP Biomedicals, Santa Ana, CA) and frozen in 10% DMSO/25% heat inactivated (HI) human AB serum (Mediatech, Manassas, VA). Fresh PBMCs isolated from de-identified buffy coats were used for fluorescence-activated cell sorting of monocytes.

### Flow cytometric analysis of protein expression

Flow cytometry on frozen PBMCs was performed as follows. Following thawing, cells (5-10x10^6^) were stained with LIVE/DEAD Fixable Aqua Dead Cell Stain (Life Technologies, Eugene, OR). Staining with unlabeled HLA-DP antibody B7/21.1 was done first, followed by a secondary BV605-labeled goat anti-mouse IgG antibody (Biolegend, San Diego, CA). The B7/21.1 antibody recognizes human HLA-DP [12, 13]. Subsequently, staining with directly labeled antibodies was performed. A cocktail of antibodies against CD3, CD19, CD56, and CD66b, all labeled with PercpCy5.5, was used to exclude T-cells, B-cells, NK cells, and neutrophils respectively (‘dump’); antibodies against CD1c and CD141 were used to identify and exclude dendritic cells, which like monocytes express CD14 and CD16. CD14-Pacific Blue (clone M5E2), and CD16-PE Cy7 (clone 3G8) were used to identify monocytes and their three subsets. Monomorphic antibody against HLA-DR (clone L243), conjugated with APC Cy7 [14] and HLA-DQ (clone 1a3), conjugated with PE (Leinco Technologies, Fenton, MO) were used, together with CerCLIP-FITC (BD Biosciences, San Jose, CA), which recognizes human CLIP bound to MHCII [15]. Following surface staining, cells were fixed with BD Cytofix and permeabilized with 1x BD Perm/Wash (BD Biosciences). Finally, the PBMCs were incubated with Map.DM1, an antibody against HLA-DM [16] conjugated to Alexa Fluor 647. Thirty PBMC samples were analyzed for HLA-DR, HLA-DP and HLA-DM; HLA-DQ was added to the last 10 samples. CLIP staining was performed in 23 samples.

Eight PBMC samples were stained with the unlabeled antibody 14–23, which binds to human HLA-DR1-CLIP, HLA-DR3-CLIP, and HLA-DR4-CLIP complexes [[17]; E. Mellins, unpublished data]: a secondary BV605-labeled goat anti-mouse IgG antibody was used. Although 14–23 is known to recognize human HLA-DR1/CLIP, HLA-DR3/CLIP, and HLA-DR4/CLIP complexes, other alleles have not been tested and may also be recognized as reactivity with the 3 tested alleles implicates binding to HLA-DR alpha, which is shared by all HLA-DR alleles. Cytokine receptors were analyzed in 10 PBMC samples; samples were stained with antibodies against CD116-FITC (GM-CSFR alpha chain, clone 4H1) and anti-CD124-APC (IL-4R alpha chain, clone G077F7).

Except as specified, all antibodies are from Biolegend. Fluorescence minus one (FMO) were used a negative control; in addition, stain with secondary antibody only (BV605-labeled goat anti-mouse IgG antibody) was used to determine level of unspecific staining. Data were collected on a Cytek DPX10 FACS machine (Cytek Biosciences, Fremont, CA). Data analysis was performed using FlowJo software (Tree Star, Ashland, OR).

### Fluorescent-activated cell sorting of monocytes

Flow cytometry based sorting was performed on freshly collected buffy coat blood samples. PBMCs were isolated by density gradient centrifugation as above, and the cells were enriched for all monocytes using the Pan Monocyte Isolation Kit (Miltenyi Biotech, San Diego, CA). Enriched monocytes were stained for surface antigens as previously described. Sterile flow cytometry sorting was performed using a BD FACSAria II (BD Biosciences) at the Stanford Shared FACS Facility (SSFF) using a 100uM nozzle, yielding monocyte subset purity of over 95% (using the classical subset). Monocyte subsets from 7 different subjects were isolated; due to limitation in cell numbers, not all stimulations were performed in all samples; specific numbers of samples tested for each stimulation are given below.

### Cytokine stimulation assays

Sorted monocytes (20,000 per test) were stimulated with various cytokines in RPMI 1640 media with 10% HI AB serum, 1% Penicillin/Streptomycin, and 1% glutamine. Monocyte subsets were left unstimulated or stimulated with 5 ng/ml of IFNγ (BioLegend, n = 6), 10 ng/ml of IL-4 (Peprotech, Rocky Hill, NJ, n = 7), 800 IU/ml of granulocyte-macrophage colony-stimulating factor (GM-CSF; Sargramostim; Immunex, Seattle, WA, n = 7), or 100 ng/ml of IL-10 (BioLegend, n = 6) for 20-24h at 37°C. Following stimulation, cells were washed with media and promptly analyzed for protein expression using flow cytometry as described above. Positively isolated CD14+ (100.000 per test) were isolated using CD14+ microbeads (Miltenyi Biotec, San Diego, CA) from 7 previously frozen PBMC samples, stimulated with IFNγ and analyzed for protein expression using flow cytometry as above.

### Statistical analyses

All statistical procedures were performed with GraphPad Prism Version 7.03 for Windows (GraphPad Software, LA Jolla, CA, www.graphpad.com). Staining data from PBMCs were tested for normality using D'Agostino & Pearson normality test and the appropriate test was then applied. For normally distributed groups we used paired one-way ANOVA without assuming equal variability of differences (sphericity) and Holm-Sidak's multiple comparisons test for group to group comparison. For groups not normally distributed we used the Friedman test, and group to group comparisons were made using the Dunn’s Multiple Comparisons test. Data from sorted monocytes and positively isolated CD14+ monocytes were analyzed using Wilcoxon matched-pairs signed rank test. Bivariate scatterplots were analyzed using the Spearman correlation test.

## Results

### Surface expression levels of MHC class II molecules, HLA-DR, -DQ and -DP, are highest in intermediate monocytes

**Fig 1:**
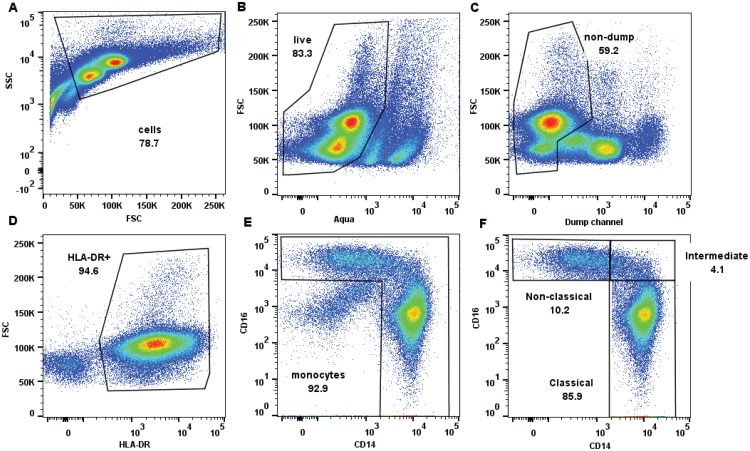
Flow cytometry gating strategy to identify the three monocyte subsets from PBMCs. (A) FSC x SSC gating to obtain mononuclear cells based on size and granularity. (B) Live/dead Aqua marker used to identify live cells (Aqua negative cells). (C) Staining with lineage-specific antibodies against T-cells, B-cells, NK cells, Dendritic cells and neutrophils (‘dump’, see Methods) to allow exclusion of these cells. (D) HLA-DR was used to identify monocytes, which are HLA-DR positive. (E) CD14 x CD16 gating used to select monocytes. (F) Gates for monocyte subsets based on CD14 and CD16 expression were determined using respective Fluorescence minus one (FMO) controls.

**Fig 2:**
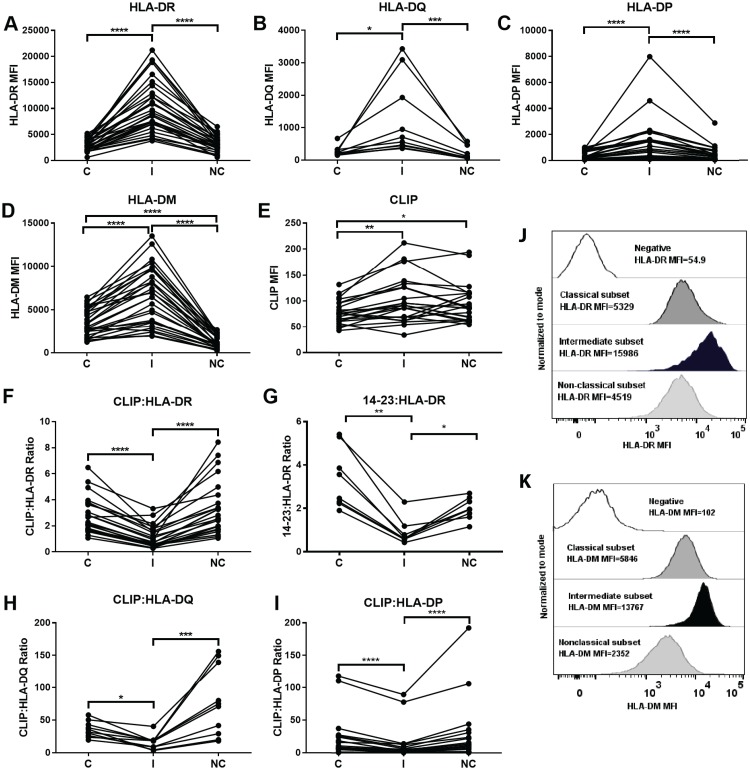
The intermediate subset expresses the highest levels of MHC class II surface protein expression and HLA-DM, and the lowest levels of CLIP to MHC class II ratio. (A) Median fluorescence intensity (MFI) of HLA-DR on the cell surface of 30 samples using L243 monoclonal antibody. (B) MFI of HLA-DQ on the cell surface of 10 samples using 1a3 monoclonal antibody. (C) MFI of HLA-DP on the cell surface of 30 samples using B7/21.1 antibody. (D) MFI of HLA-DM using Map.DM1 antibody after permeabilization on 30 samples. (E) MFI of surface CLIP using CerCLIP antibody on 23 samples. (F) CLIP:HLA-DR ratio was calculated by dividing the surface CerCLIP MFI by the surface HLA-DR MFI for 23 samples. (G) The 14–23:HLA-DR ratio was calculated by dividing the surface 14–23 MFI by the surface HLA-DR MFI for 8 samples. (H) CLIP:HLA-DQ ratio was calculated by dividing the surface CerCLIP MFI by the surface HLA-DQ MFI for 10 samples. (I) CLIP:HLA-DP ratio was calculated by dividing the surface CerCLIP MFI by the surface HLA-DP MFI for 23 samples. CLIP:MHCII and 14–23:HLA-DR ratios are multiplied by 100. (J) Representative histogram of flow cytometry staining for HLA-DR. (K) Representative histogram of flow cytometry staining for HLA-DM. In (J) and (K), Fluorescence minus one (FMO) was used as negative control. Monocyte subsets were paired for each individual sample, thus statistical analysis used Friedman tests with Dunn’s Multiple Comparison tests, except for (G) and (H) were Wilcoxon matched-pairs signed rank test was used for group to group comparison. Statistical significance represented by asterisk: *, p < 0.05; **, p < 0.01; ***, p < 0.001; ****, p <0.0001. C: Classical; I: Intermediate; NC: Non-classical.

To begin to analyze class II pathway constituents in human monocyte subsets, we measured surface expression levels of the MHCII molecules, HLA-DR, -DP, and –DQ, on the 3 monocyte subsets within PBMC from healthy adults; Fig 1 illustrates the gating strategy used to identify the monocyte subsets. We found that intermediate monocytes expressed the highest level of surface HLA-DR, whereas expression levels were lower and similar between the classical and non-classical monocytes (Fig 2A and 2J). Surface HLA-DQ and -DP also were expressed at higher levels in the intermediate subset and at comparable, lower levels in the classical and non-classical monocytes (Fig 2B and 2C).

### HLA-DM expression is highest, and CLIP:MHCII ratios lowest, in intermediate monocytes

We found that intermediate monocytes had the highest levels of HLA-DM; the lowest levels were found in non-classical monocytes (Fig 2D and 2K). To evaluate differences in HLA-DM functionality between the monocyte subsets, we measured levels of CLIP at the cell surface. We observed that, among the 3 subsets, total surface levels of CLIP were lowest in the classical subset (Fig 2E). To evaluate the efficiency of CLIP exchange for other peptides, levels of CLIP must be normalized for levels of class II proteins. CLIP:HLA-DR ratios were calculated from the flow cytometry data by dividing the CerCLIP median fluorescence intensity (MFI) by the L243 (anti-HLA-DR) MFI. In accordance with higher levels of HLA-DR and HLA-DM in intermediate monocytes, this subset showed the lowest CLIP:HLA-DR ratio levels compared to classical and non-classical subsets (Fig 2F). The CLIP:HLA-DR ratio was not significantly different between the classical and non-classical monocytes, despite the difference in HLA-DM levels between these 2 subsets (Fig 2D and 2K). As a more precise measure of CLIP loading onto surface HLA-DR, we used the 14–23 monoclonal antibody, which specifically binds HLA-DR/CLIP complexes, whereas the CERCLIP antibody binds to CLIP loaded onto any class II molecule. The intermediate monocyte subset had the lowest HLA-DR-CLIP (14–23):HLA-DR (L243) ratios (Fig 2G), corroborating the CLIP:HLA-DR ratio findings.

**Fig 3:**
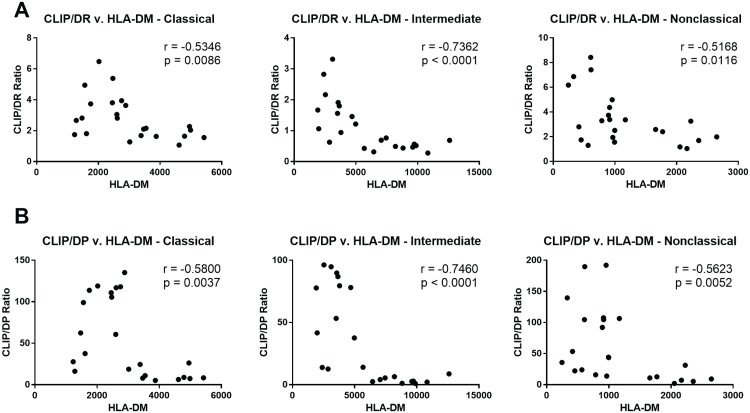
CLIP:DR and CLIP:DP ratios correlate negatively with HLA-DM expression in monocyte subsets. (A) Spearman correlation between the CLIP:HLA-DR ratios and HLA-DM MFI’s for the three monocyte subsets from 23 samples. (B) Spearman correlation between the CLIP:HLA-DP ratios and HLA-DM MFIs for the three monocyte subsets from 23 samples. All CLIP:MHCII ratios are multiplied by 100.

We also determined CLIP:HLA-DQ and CLIP:HLA-DP ratios using the CerCLIP antibody. Similar to CLIP:HLA-DR, CLIP:HLA-DQ and CLIP:HLA-DP ratios were significantly lower for intermediate monocytes as compared to the classical and non-classical subsets (Fig 2H and 2I). Using Spearman correlation, we found that the CLIP:HLA-DR ratio correlated negatively with HLA-DM expression, achieving statistical significance in all three monocyte subsets (Fig 3A). A negative correlation also was found for the CLIP:HLA-DP ratio and HLA-DM expression (Fig 3B). The negative correlations were the strongest in the intermediate monocytes. Negative correlations between HLA-DM and CLIP:HLA-DQ were also observed for the 3 subsets (S1 Fig), with the non-classical subset showing the strongest correlation.

### Cytokine stimulation of sorted monocyte subsets

**Fig 4:**
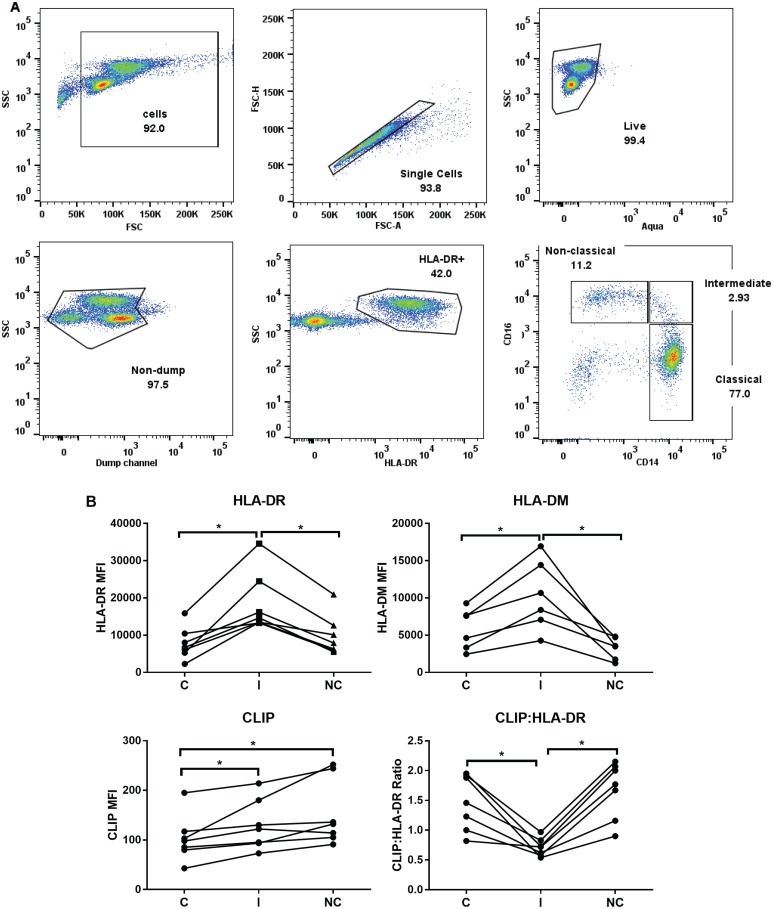
Profile of sorted, unstimulated monocytes in culture for 20–24 h is similar to monocytes within PBMC. (A) Flow cytometry gating scheme depicting the gating strategy used to identify the three monocyte subsets for sorting. Isolated PBMCs, enriched for monocytes (see Methods) were gated on a FSC x SSC plot; single, live cells were selected. Cells negative for neutrophils, and T, B, NK and Dendritic cells were further selected (‘non-dump’), and HLA-DRxSSC was used to identify monocytes (HLA-DR+). Gates for monocyte subsets based on CD14 and CD16 expression were determined using respective FMO controls. (B) Median fluorescence intensity (MFI) of surface HLA-DR, HLA-DM, surface CLIP and CLIP:HLA-DR ratios from 7 individual sorted samples (except HLA-DM, n = 6). Wilcoxon matched-pairs signed rank test was used for group to group comparison. Statistical significance represented by asterisk: *, p < 0.05. C: Classical; I: Intermediate; NC: Non-classical.

We next investigated the patterns of HLA-DR, HLA-DM and CLIP expression in the 3 monocyte subsets in response to several cytokine stimuli. We used sorted monocyte subsets and focused on HLA-DR, the most abundant human class II isotype, for these studies; Fig 4A shows the gating strategy used for sorting [5]. We observed that in unstimulated monocytes following sorting and 20-24h culture, the differences previously observed for levels of surface HLA-DR, HLA-DM, surface CLIP and CLIP:HLA-DR ratio among the different subsets were maintained (Fig 4B).

### IFNγ effects on MHCII in monocyte subsets

**Fig 5:**
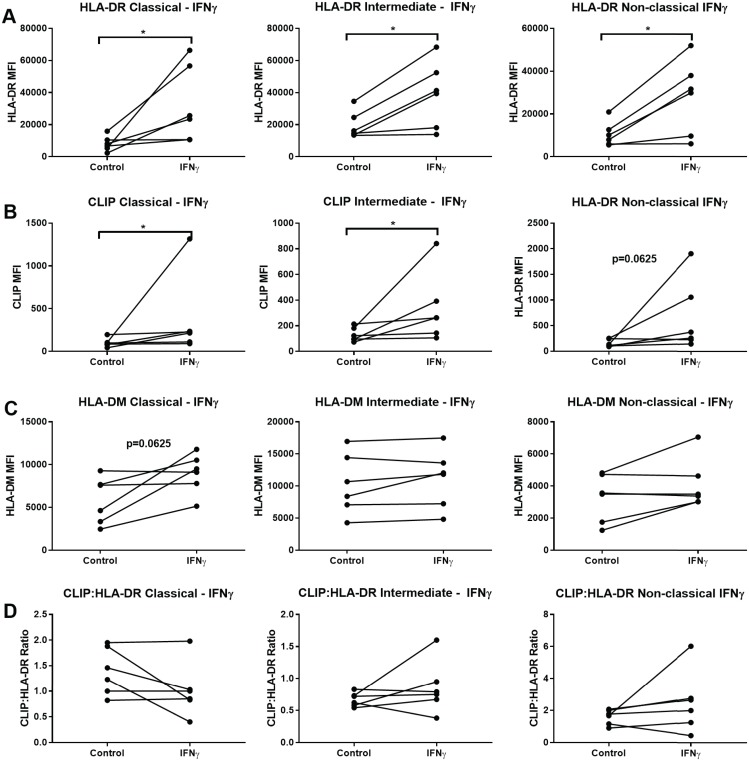
IFNγ stimulation increases HLA-DR and CLIP surface expression in all monocyte subsets. Sorted monocytes from each subset were incubated with 5 ng/ml of IFNγ for 20–24 hours or left unstimulated (control). (A) Cells were stained for surface HLA-DR using L243. Median fluorescence intensity (MFI) was obtained. (B) CLIP MFI measured using the CerCLIP antibody. (C) MFI of HLA-DM using the MapDM1 antibody. (D) CLIP:HLA-DR ratios calculating dividing the MFI of CLIP by the HLA-DR MFI and multiplying by 100. Six independent samples were tested. Wilcoxon matched-pairs signed rank test was used for group to group comparison. Statistical significance represented by asterisk: *, p < 0.05.

We observed that following IFNγ stimulation, all 3 monocyte subsets significantly upregulated expression of surface HLA-DR (Fig 5A). HLA-DR fold change was lower in the intermediate subset compared to the other subsets (S2 Fig). CLIP surface expression also was increased by IFNγ in all 3 subsets, although to a lesser degree in the non-classical subset, where 5 out of 6 pairs showed an increase, whereas for the classical and intermediate subsets, all 6 pairs showed increased CLIP expression (Fig 5B). Changes in HLA-DM expression (as detected by anti-DM dimer antibody, mapDM.1) following IFNγ stimulation were more noticeable in the classical subset, where 4 out of 6 samples showed at an average increase of 2.2x over control (unstimulated) levels (Fig 5C).

Although IFNγ stimulation increased surface expression of HLA-DR and CLIP, and to a lesser degree, the expression of HLA-DM, in a mostly consistent pattern across the 3 subsets, CLIP:HLA-DR ratios varied across and within the subsets (Fig 5D). To further elucidate the consequence of IFNγ-induction on HLA-DR, HLA-DM and surface CLIP:HLA-DR ratios, we performed additional experiments using positively isolated CD14+ monocytes from 7 previously frozen PBMC samples, representing mostly classical monocytes, where the increases were most substantial and consistent across different donors.

**Fig 6:**
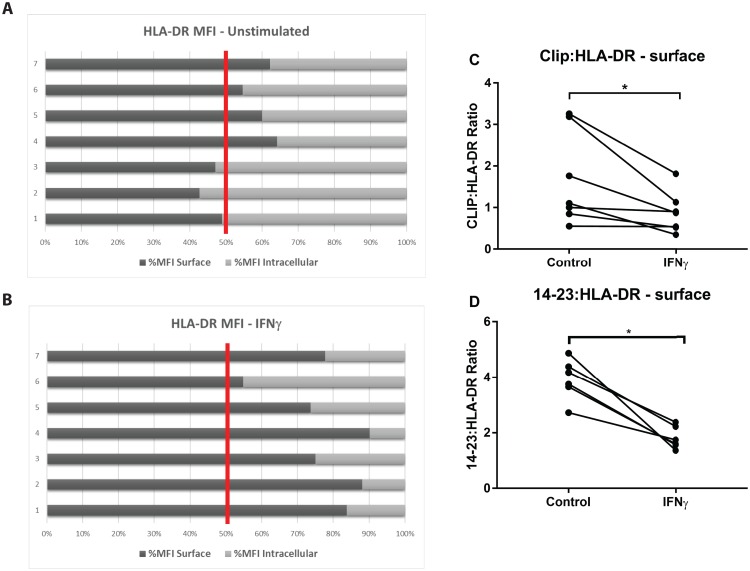
IFNγ stimulation preferentially increases the expression of HLA-DR at the surface. (A) HLA-DR expression was determined using L243 antibody in unstimulated, positively isolated CD14+ monocytes, both at the surface and intracellularly (defined as the difference between total HLA-DR MFI and the surface MFI) after 20–24 hours; MFI expressed as percentage. (B) CD14+ monocytes were stimulated with 5 ng/ml of IFNγ for 20–24 hours and HLA-DR expression determined as in (A). (C) CLIP:HLA-DR ratio was calculated by dividing the surface CerCLIP MFI by the surface HLA-DR MFI. (D) The 14-23/HLA-DR ratio was calculated by dividing the surface 14–23 MFI by the surface HLA-DR MFI. Seven samples were tested, except for the 14–23 antibody, n = 6. Wilcoxon matched-pairs signed rank test was used for group to group comparison. Statistical significance represented by asterisk: *, p < 0.05.

We found that IFNγ induces an increase of the total expression (surface+intracellular) of HLA-DR, with a higher relative increase in surface HLA-DR. Specifically, in unstimulated CD14+ monocytes, HLA-DR was found to be approximately equally distributed between surface and inside the cell (Fig 6A). Comparing the expression of HLA-DR in the surface and total following IFNγ stimulation, close to 80% of HLA-DR expression was detected in the surface (Fig 6B). CLIP expression was also increased, both at the surface and inside the cell. As observed with HLA-DR, in unstimulated cells, CLIP was close to equally distributed between intracellular and surface compartments. After IFNγ stimulation, close to 70% of CLIP was detected at the surface (S3 Fig). In 6 out of 7 (70%) samples, the CLIP:HLA-DR ratio at the surface decreased at least 10% following IFNγ stimulation (Fig 6C). To more precisely evaluate CLIP occupancy of HLA-DR, we measured changes in levels of mAb 14.23 binding in 6 of these samples. Using positively isolated CD14+, we found that the ratios of 14–23:HLA-DR at the surface were decreased in all 6 samples tested after IFNγ stimulation (Fig 6D).

### GM-CSF effects on MHCII in monocyte subsets

**Fig 7:**
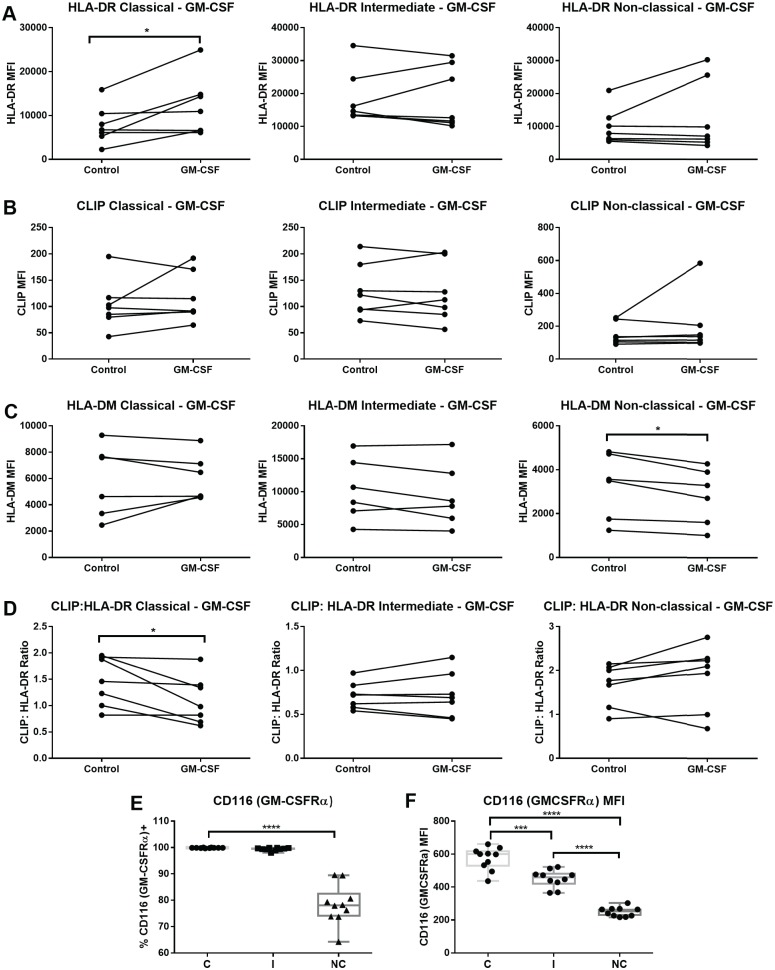
GM-CSF stimulation increases surface HLA-DR in the classical subset and decreases HLA-DM in the non-classical subset, and GM-CSF receptor alpha chain shows differential surface expression among monocyte subsets. Sorted monocytes from each subset were incubated with 800 IU/ml of GM-CSF for 20–24 hours or left unstimulated (control). (A) Median fluorescence intensity (MFI) of HLA-DR measured using the L243 antibody. (B) CLIP MFI measured using the CerCLIP antibody. (C) MFI of HLA-DM using the MapDM1 antibody. (D) CLIP:HLA-DR ratios calculating dividing the MFI of CLIP by the HLA-DR MFI and multiplying by 100. Seven independent samples were tested, except for HLA-DM (n = 6). Wilcoxon matched-pairs signed rank test was used for group to group comparison. (E) Percentage of CD116 (GM-CSFR alpha chain) positive monocytes in each subset among PBMCs. (F) CD116 (GM-CSFR alpha chain) MFI in each subset among PBMCs. Ten independent samples were tested. Repeat measures ANOVA with Holm-Sidak's multiple comparisons test was used (for E and F). C: Classical; I: Intermediate; NC: Non-classical. Statistical significance represented by asterisk: *, p < 0.05, ***, p < 0.001; ****, p <0.0001.

Different responses among the monocytes subsets were observed following stimulation with GM-CSF, which induced a significant increase of surface HLA-DR only in classical monocytes (Fig 7A). Surface CLIP expression was not changed after GM-CSF stimulation (Fig 7B). Using MapDM1 antibody, we did not observe an increase in HLA-DM in any of subsets; in fact, there is a progressive increase in the number of samples that reduce HLA-DM in response to GM-CSF from classical to non-classical subset, where the decrease is statistically significant (Fig 7C). However, we found that the CLIP:HLA-DR ratio is decreased by GM-CSF in the classical subset (Fig 7D). The non-classical subset showed CLIP:HLA-DR increase in 5/7 samples (average 1.2x); decrease of HLA-DM could be a contributing factor (Fig 7C).

Differential surface expression of cytokine receptors among the monocyte subsets is one potential factor affecting their distinct responses to GM-CSF. The GM-CSF receptor comprises 2 subunits, the ligand specific GM-CSFRα chain, and the GM-CSFβ chain, common to the GM-CSF, IL-3 and IL-5 receptors. Of interest, in the classical and intermediate subsets all cells are positive for GM-CSFRα, whereas in non-classical monocytes the expression of the ligand-specific GM-CSFRα subunit appears to further subset the group into positive and negative cells (Fig 7E). Further, the *ex-vivo* baseline expression of GM-CSFRα varies with classical > intermediate > non-classical, on the expressing cells (Fig 7F).

### IL-4 effects on MHCII in monocyte subsets

**Fig 8:**
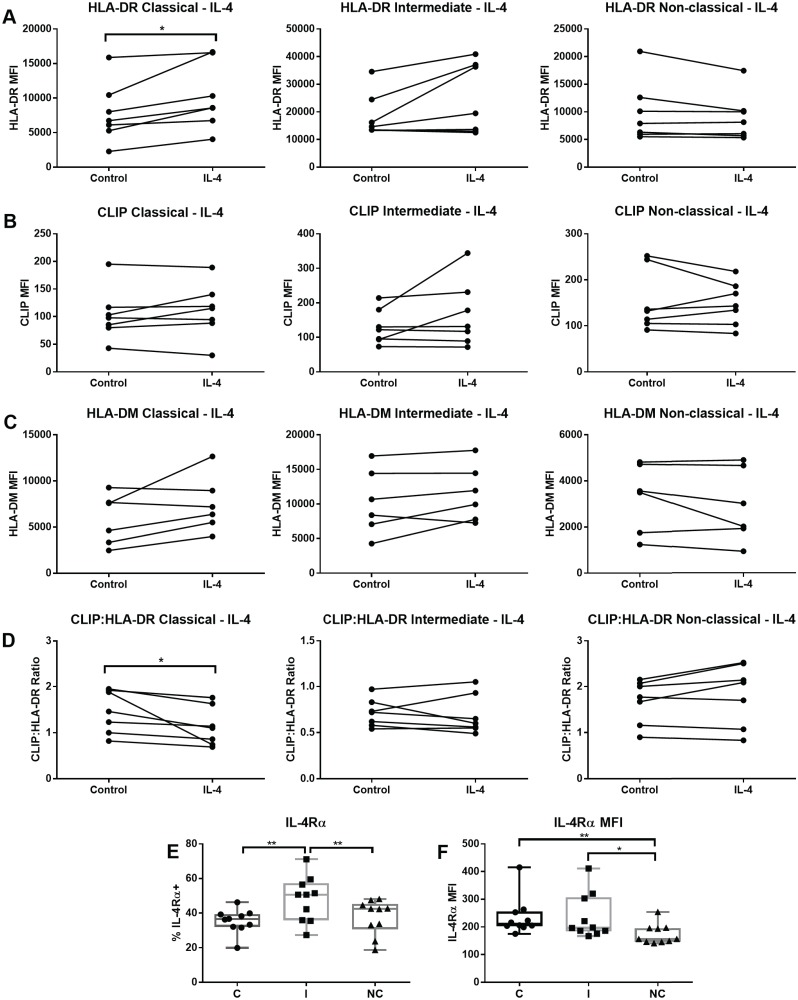
IL-4 stimulation increases HLA-DR surface expression in the classical subset, and IL-4 receptor alpha chain is differentially expressed among monocyte subsets. Sorted monocytes from each subset were incubated with 10 ng/ml of IL-4 for 20–24 hours or left unstimulated (control). (A) Median fluorescence intensity (MFI) of HLA-DR measured using the L243 antibody. (B) Median fluorescence intensity (MFI) of CLIP measured using the CerCLIP antibody. (C) Median fluorescence intensity (MFI) of HLA-DM using the MapDM1 antibody. (D) CLIP:HLA-DR ratios calculating dividing the MFI of CLIP by the HLA-DR MFI and multiplying by 100. Seven independent samples were tested, except for HLA-DM (n = 6). Wilcoxon matched-pairs signed rank test was used for group to group comparison. (E) Percentage of CD124 (IL-4R alpha chain) positive monocytes in each subset among PBMCs. (F) CD124 (IL-4R alpha chain) MFI in in each subset among PBMCs. Ten independent samples were tested. Repeat measures ANOVA with Holm-Sidak's multiple comparisons test was used (for E and F). C: Classical; I: Intermediate; NC: Non-classical. Statistical significance represented by asterisk: *, p < 0.05, **, p < 0.01.

IL-4 exposure induced a significant increase in surface HLA-DR in the classical subset and in 4/6 samples of the intermediate subset (average increase 1.6x, Fig 8A) In contrast, HLA-DR on the non-classical monocytes was reduced or unchanged. IL-4 stimulation induced no observable changes in surface CLIP expression (Fig 8B), and no significant changes for HLA-DM, except in the classical subset, which showed a trend for increased HLA-DM, with 4/6 samples increasing HLA-DM (average increase 1.6x, Fig 8C), and decreased CLIP:HLA-DR ratio after IL-4 (Fig 8D). We measured expression of surface IL-4Rα, the ligand binding chain of the IL-4 receptor, and observed positive and negative subgroups within each of the defined monocyte subsets. The intermediate subset had the highest percentage cells expressing IL-4Rα, and the non-classical subset had the lowest level of IL-4Rα expression (Fig 8E and 8F).

### IL-10 effects on MHCII in monocyte subsets

**Fig 9:**
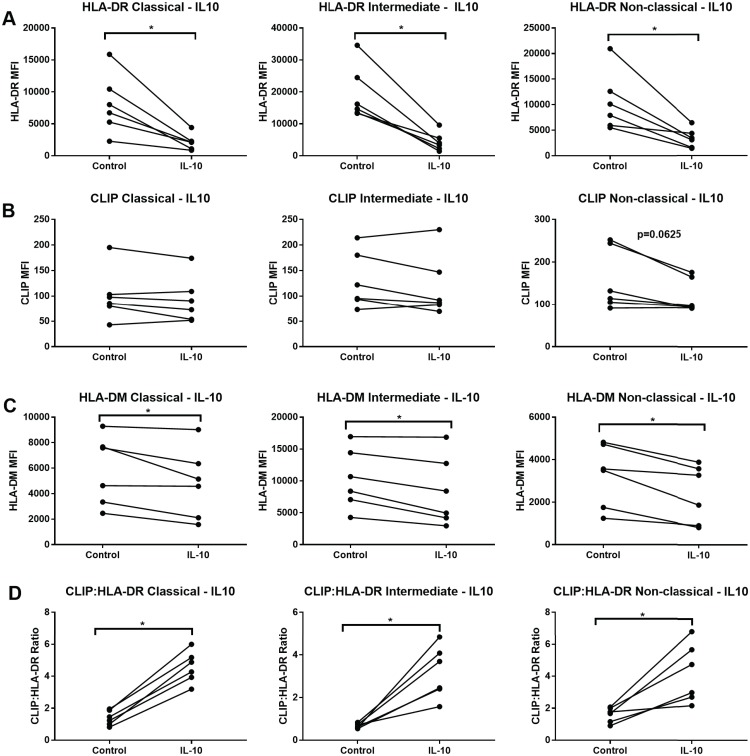
IL-10 stimulation decreases HLA-DR surface expression in all 3 monocyte subsets. Sorted monocytes from each subset were incubated with 100 ng/ml of IL-10 for 20–24 hours or left unstimulated (control). (A) Cells were stained for surface HLA-DR using L243. Median fluorescence intensity (MFI) was obtained. (B) CLIP MFI measured using the CerCLIP antibody. (C) MFI of HLA-HLA-DM using the MapDM1 antibody. (D) CLIP:HLA-DR ratios calculating dividing the MFI of CLIP by the HLA-DR MFI and multiplying by 100. Six independent samples were tested. Wilcoxon matched-pairs signed rank test was used for group to group comparison. Statistical significance represented by asterisk: *, p < 0.05.

Incubation with IL-10 significantly reduced the HLA-DR expression in all 3 monocyte subsets (Fig 9A). CLIP surface expression was not significantly altered after IL-10 stimulation, although there was a trend for decreased surface CLIP expression in the non-classical subset (Fig 9B). IL-10 also induced a significant decrease of HLA-DM expression (Fig 9C) and a significant increase of CLIP:HLA-DR in all 3 subsets (Fig 9D). Overall, the 3 monocyte subsets showed a largely similar pattern of responses to IL-10.

## Discussion

Our study shows that the human 3 monocyte subsets, identified based on CD14 and CD16 expression, have distinct expression patterns of MHCII molecules and HLA-DM at steady state and that these molecules are modulated differently in response to cytokine stimulation in the different subsets. These differences likely reflect distinct functions of these subsets during homeostasis and in response to cytokine environments.

When analyzing monocytes within unstimulated PBMCs, we observed that the intermediate monocyte subset expresses the highest level of surface HLA-DR, in accordance with previous reports [7, 18]. We found that the intermediate monocytes also express the highest surface level of HLA-DP and HLA-DQ proteins, consistent with gene expression studies that showed that HLA-DPA1, -DPB1, and -DQB1 transcripts are elevated in intermediate monocytes [7, 8].

Among the 3 monocyte subsets, intermediate monocytes also express the highest level of HLA-DM, the MHCII peptide exchange catalyst. HLA-DM catalyzes exchange of CLIP for other peptides on class II molecules. In the absence of HLA-DM function, most MHCII alleles are expressed at the cell surface with CLIP still bound. To measure total surface CLIP, we used the CerCLIP antibody, which binds to the CLIP N-terminal region, which extends beyond the peptide binding groove [15]; thus, CerCLIP binds to CLIP bound to any MHCII allele. In line with the high expression of HLA-DM, intermediate monocytes also have the lowest CLIP:MHCII and DR-CLIP(14.23):HLA-DR ratios of all 3 subsets, an indirect measure of HLA-DM function. HLA-DM expression negatively correlates with CLIP:MHC ratio, suggesting that the level of HLA-DM is primarily responsible for the levels of CLIP on the cell surface, at least for the intermediate subset. These findings are consistent with the known function of HLA-DM. However, the relationship between the levels of HLA-DM and CLIP:MHCII can be modulated by MHCII allelic affinity for CLIP or for HLA-DM. This and other factors [19] may contribute to the wide distribution of CLIP:MHCII ratios at lower levels of HLA-DM expression, where the affinity differences will be the most consequential. It has been suggested that the intermediate subset possesses the highest capacity to present antigen [6], and it has been shown that the intermediate subset induces the highest proliferation in CD4+ T cells stimulated with SEB [8]. Our results provide insight into the possible mechanisms involved in this phenotype. Our findings that CLIP:MHCII and HLA-CLIP(14.23):HLA-DR ratios are lowest on intermediate monocytes imply that this subset presents other peptide/MHCII complexes efficiently. In addition to HLA-DM, other factors may contribute to the enhanced capacity for class II restricted antigen presentation, such as levels of endosomal proteases, which generate peptides for MHC II presentation. For example, cathepsins B and L have been shown to be elevated in intermediate monocytes [8, 20]. However, it was also reported that intermediate monocytes can inhibit autologous proliferation and induce production of IL-10 by CD4+ T cells [21]. Thus, the functional consequence of interaction with CD4+ T cells may differ, depending on the activation state or polarization of the intermediate subset. Expression of co-stimulatory molecules have also been reported to be elevated in intermediate monocytes, such as CD40 [7], and CD86, although CD86 has also been found to be elevated in non-classical monocytes [18, 22]. The mode of isolation of monocytes may also play a role on the phenotype of monocytes [18].

We expected to see lower CLIP:HLA-DR ratios in the classical compared to the non-classical subset, given that HLA-DR levels are generally comparable whereas HLA-DM levels are generally increased in the classical subset compared to the non-classical monocytes. Higher levels of binding of the 14–23 antibody were observed on classical monocytes, raising the possibility that other peptides in addition to CLIP may generate the conformation recognized by mAb 14–23, although this antibody was generated by immunization of HLA-DR3 transgenic mice with HLA-DR3/CLIP complexes [17]. Interestingly, the classical monocyte has been observed to be the most efficient at inducing autologous T cell proliferation, although by a small degree [21, 23]. These results suggest that although the intermediate subset has the highest level of MHCII, the lowest CLIP:MHCII ratio, and high levels of costimulatory molecules [7], the classical subset may present relevant self-peptides for homeostatic proliferation [24]. Monocytes plus IL-7 are sufficient to induce homeostatic proliferation of CD4+ T cells *in vitro* [25]. Furthermore, CLIP:MHCII peptide complexes appear to be involved in homeostatic proliferation of naïve T cells [24].

Compared to the classical and intermediate subsets, the non-classical subset showed lower levels of expression of MHCII-related molecules and a decreased response to activating cytokines (see below). The non-classical subset has been shown to be involved in patrolling of the endothelial layer of blood vessels [26], and their role in antigen presentation may be secondary to their patrolling functions.

When the isolated monocyte subsets were stimulated with different cytokines, the classical subset appeared to be the main responder for activating cytokines (IFNγ, GM-CSF and IL-4). Following stimulations with these cytokines, the classical subset showed increased surface expression of HLA-DR and decreased ratios of CLIP:HLA-DR, implying a recruitment of this subset into an antigen presentation role. Although the intermediate and the non-classical subsets also increased expression of surface HLA-DR in response to IFNγ, the classical subset showed more robust fold changes. The classical subset also showed more consistent changes in CLIP:HLA-DR ratios and a moderate increase in HLA-DM following IFNγ stimulation. IFNγ is a potent activator of monocytes and is known to upregulate MHCII expression [27]. IFNγ is also a key cytokine for polarization of monocytes towards an M1, or classically activated phenotype, associated with production of pro-inflammatory mediators [note: the classification of monocyte polarization types is under discussion and will probably be revised in the future [28, 29]]. CLIP expression was also increased in all 3 subsets, although the level of increase varied between individual donors, possibly a reflection of differences in the MHCII alleles; the CLIP increase is consistent with previous reports showing that the invariant chain is induced by IFNγ [30]; additionally, proteases that may be involved in cleavage of Ii have also been shown to be induced by IFNγ [31].

Increased HLA-DR and decreased CLIP:HLA-DR ratio in response to GM-CSF were observed only in the classical subset, confirming our previous observation using total CD14+ monocytes [11]. By resolving the subsets, we now show the decrease occurs in the classical subset. GM-CSF is another pro-inflammatory and M1-inducing cytokine [32]; [the terminology will likely change [29]]. GM-CSF has been shown to increase monocyte surface expression of HLA-DR *in vivo*, in patients with sepsis receiving GM-CSF, and this increase could contribute to countering the immunosuppression associated with sepsis [33]. Furthermore, GM-CSF treatment increased expression of HLA-DR on monocytes in immunosuppressed patients after surgery; GM-CSF treated group had fewer days with infection compared to placebo and influenza vaccination as a treatment [34]. A subsequent analysis of this trial showed that, in addition to increased HLA-DR, monocytes from GM-CSF treated patients showed increased release of LPS-induced TNF [35]. These results suggest a potentially clinically relevant role for the GM-CSF induction of HLA-DR.

The CLIP:HLA-DR ratios decreased following GM-CSF in the classical subset. Given the lack of alteration in HLA-DM levels, this result may reflect improved intracellular co-localization of HLA-DR and HLA-DM after GM-CSF treatment of classical monocytes, suggesting recruitment of this subset into an antigen presentation role also in the context of GM-CSF-associated inflammation. The finding that the classical subset shows the highest levels of expression of the ligand binding GM-CSF alpha chain among the 3 subsets may contribute to its apparently enhanced response to GM-CSF.

IL-4 is a Th2 cytokine and activates monocytes to an alternatively-activated, M2-phenotype; this phenotype is generally associated with anti-inflammatory responses. With IL-4 stimulation, the classical subset was again the main responder, with the intermediate subset showing a similar pattern, albeit to a reduced degree. Non-classical monocytes did not show a detectable response to IL-4. This result is consistent with other studies, which found that non-classical monocytes have a reduced cytokine response to IL-4 [23]. Differences between CD16+ and CD16- monocytes in induction of some cell markers during dendritic cell differentiation by GM-CSF, IL-4 and TNFα have also been reported [36]. Levels of IL4Rα were similar between classical and intermediate subsets, with non-classical monocytes showing the lowest levels, which may contribute to the lack of response from the non-classical subset.

The mechanisms underlying the changes in MHCII molecules expression induced by cytokines likely involve several processes, including transcription and translation, as well as post-translation events such as intracellular trafficking. Using CD14+ monocytes and IFNγ, a combination that showed the most robust responses, we found that IFNγ not only induces the expression of HLA-DR and CLIP, but it appears to induce a preferential trafficking of these molecules to the cell surface. It is also possible that IFNγ increases longevity of HLA-DR molecules at the surface. IFNγ has been shown to be involved in stability of class I molecules [37], and similar mechanisms could be involved in class II molecules. Kinetics of induction of HLA-DR and HLA-DM may also be different [38], and the molecules may not encounter each other well, introducing further variability in CLIP:HLA-DR ratios.

IL-10 is an anti-inflammatory cytokine that has been found to be a strong suppressor of immune function. IL-10 is thought to mediate anti-inflammatory and immunosuppressive actions of monocytes at least in part by down-regulating surface expression of MHCII molecules [39]. Accordingly, and in contrast to the stimulatory cytokines, IL-10 exposure results in large decreases of surface HLA-DR expression, and decreases in HLA-DM expression in all 3 subsets. The CLIP:HLA-DR ratio was also decreased in all 3 subsets, indicating that reduction in antigen presentation role in all monocytes in the IL-10 microenvironment.

Our results add to the evidence that the intermediate subset, at baseline, is better equipped to present antigen, although their precise role as APC during homeostasis is still unclear. During activating conditions, classical monocytes appear to be preferentially recruited into an antigen presenting role. This capacity to respond to activating stimuli may be relevant to the role of classical monocytes at sites of inflammation. Data from animal models as well as human studies indicate that classical monocytes may be the first subset recruited into sites of inflammation, where they encounter a highly inflammatory environment and where their roles include activation of immune cells and production of proinflammatory mediators. Intermediate monocytes may appear after classical monocytes and may also participate in antigen presentation, production of proinflammatory mediators as well as angiogenesis. Non-classical monocytes may arrive later and be associated with wound healing [40].

Down-regulation of monocyte antigen presentation function in all three monocyte subsets is accomplished by IL-10, perhaps physiologically during resolution of inflammation. Notably, pathogens, including Epstein-Barr virus and cytomegalovirus, make IL-10 homologues, apparently as an immune evasion strategy to reduce class II-restricted presentation of viral antigen by monocytes [41, 42].

Perturbations in the frequency of intermediate as well as non-classical monocytes have been observed in a variety of conditions, including autoimmune diseases, bacterial and viral infections and several inflammatory conditions such as asthma, coronary artery disease, periodontitis and stroke [6, 43]. Further characterization of monocytes subsets, including improved ways to discriminate among the monocyte subsets [44], will help understand the role of these cells in health as well as in disease.

## Supporting information

S1 Fig CLIP/DQ ratio correlate negatively with HLA-DM expression in monocyte subsets. Spearman correlation between the CLIP/DQ ratios (multiplied by 100) and HLA-DM MFI’s for the three monocyte subsets from 10 samples.
(PDF)

S2 Fig HLA-DR fold change is lowest in the intermediate subset. Sorted monocytes from each subset were incubated with 5 ng/ml of IFNγ for 20–24 hours or left unstimulated. Cells were stained for surface HLA-DR using L243, and median fluorescence intensity (MFI) was obtained. Fold change for HLA-DR was obtained by dividing the HLA-DR MFI after IFNγ stimulation by the MFI of the unstimulated subset. Six independent samples were tested. Wilcoxon matched-pairs signed rank test was used for group to group comparison. Statistical significance represented by asterisk: *, p < 0.05. C: Classical; I: Intermediate; NC: Non-classical.
(PDF)

S3 Fig IFNγ stimulation preferentially increases the expression of CLIP at the surface. (A) CLIP expression was determined using CerCLIP antibody in unstimulated, positively isolated CD14+ monocytes, both at the surface and intracellularly (defined as the difference between total CLIP MFI and the surface MFI) after 20–24 hours; MFI expressed as percentage. (B) CD14+ monocytes were stimulated with 5 ng/ml of IFNγ for 20–24 hours and CLIP expression determined as in (A).
(PDF)

## References

[1] Auffray C, Sieweke MH, Geissmann F. Blood monocytes: development, heterogeneity, and relationship with dendritic cells. Annual review of immunology. 2009;27:669–92. Epub 2009/01/10. doi: 10.1146/annurev.immunol.021908.132557 . 19132917 10.1146/annurev.immunol.021908.132557

[2] Italiani P, Boraschi D. From Monocytes to M1/M2 Macrophages: Phenotypical vs. Functional Differentiation. Front Immunol. 2014;5:514 Epub 2014/11/05. doi: 10.3389/fimmu.2014.00514 . 25368618 10.3389/fimmu.2014.00514 PMC4201108

[3] Jakubzick CV, Randolph GJ, Henson PM. Monocyte differentiation and antigen-presenting functions. Nat Rev Immunol. 2017;advance online publication. doi: 10.1038/nri.2017.28 http://www.nature.com/nri/journal/vaop/ncurrent/abs/nri.2017.28.html#supplementary-information. 28436425 10.1038/nri.2017.28

[4] Ginhoux F, Jung S. Monocytes and macrophages: developmental pathways and tissue homeostasis. Nat Rev Immunol. 2014;14(6):392–404. doi: 10.1038/nri3671 24854589 10.1038/nri3671

[5] Ziegler-Heitbrock L. Blood Monocytes and Their Subsets: Established Features and Open Questions. Front Immunol. 2015;6:423 doi: 10.3389/fimmu.2015.00423 . 26347746 10.3389/fimmu.2015.00423 PMC4538304

[6] Wong KL, Yeap WH, Tai JJ, Ong SM, Dang TM, Wong SC. The three human monocyte subsets: implications for health and disease. Immunol Res. 2012;53(1–3):41–57. doi: 10.1007/s12026-012-8297-3 . 22430559 10.1007/s12026-012-8297-3

[7] Wong KL, Tai JJ, Wong WC, Han H, Sem X, Yeap WH, et al Gene expression profiling reveals the defining features of the classical, intermediate, and nonclassical human monocyte subsets. Blood. 2011;118(5):e16–31. doi: 10.1182/blood-2010-12-326355 . 21653326 10.1182/blood-2010-12-326355

[8] Zawada AM, Rogacev KS, Rotter B, Winter P, Marell RR, Fliser D, et al SuperSAGE evidence for CD14++CD16+ monocytes as a third monocyte subset. Blood. 2011;118(12):e50–61. doi: 10.1182/blood-2011-01-326827 . 21803849 10.1182/blood-2011-01-326827

[9] Hijdra D, Vorselaars AD, Grutters JC, Claessen AM, Rijkers GT. Phenotypic characterization of human intermediate monocytes. Front Immunol. 2013;4:339 doi: 10.3389/fimmu.2013.00339 . 24155746 10.3389/fimmu.2013.00339 PMC3805031

[10] Busch R, Rinderknecht CH, Roh S, Lee AW, Harding JJ, Burster T, et al Achieving stability through editing and chaperoning: regulation of MHC class II peptide binding and expression. Immunological reviews. 2005;207:242–60. Epub 2005/09/27. doi: 10.1111/j.0105-2896.2005.00306.x . 16181341 10.1111/j.0105-2896.2005.00306.x

[11] Hornell TM, Beresford GW, Bushey A, Boss JM, Mellins ED. Regulation of the class II MHC pathway in primary human monocytes by granulocyte-macrophage colony-stimulating factor. J Immunol. 2003;171(5):2374–83. . 12928384 10.4049/jimmunol.171.5.2374

[12] Watson AJ, DeMars R, Trowbridge IS, Bach FH. Detection of a novel human class II HLA antigen. Nature. 1983;304(5924):358–61. Epub 1983/07/03. . 6192342 10.1038/304358a0

[13] Mellins E, Smith L, Arp B, Cotner T, Celis E, Pious D. Defective processing and presentation of exogenous antigens in mutants with normal HLA class II genes. Nature. 1990;343(6253):71–4. doi: 10.1038/343071a0 . 1967485 10.1038/343071a0

[14] Fu XT, Karr RW. HLA-DR alpha chain residues located on the outer loops are involved in nonpolymorphic and polymorphic antibody-binding epitopes. Hum Immunol. 1994;39(4):253–60. . 7520896 10.1016/0198-8859(94)90268-2

[15] Ghosh P, Amaya M, Mellins E, Wiley DC. The structure of an intermediate in class II MHC maturation: CLIP bound to HLA-DR3. Nature. 1995;378(6556):457–62. Epub 1995/11/30. doi: 10.1038/378457a0 . 7477400 10.1038/378457a0

[16] Denzin LK, Cresswell P. HLA-DM induces CLIP dissociation from MHC class II alpha beta dimers and facilitates peptide loading. Cell. 1995;82(1):155–65. Epub 1995/07/14. . 7606781 10.1016/0092-8674(95)90061-6

[17] Stang E, Guerra CB, Amaya M, Paterson Y, Bakke O, Mellins ED. DR/CLIP (class II-associated invariant chain peptides) and DR/peptide complexes colocalize in prelysosomes in human B lymphoblastoid cells. J Immunol. 1998;160(10):4696–707. Epub 1998/05/20. . 9590215

[18] Mukherjee R, Kanti Barman P, Kumar Thatoi P, Tripathy R, Kumar Das B, Ravindran B. Non-Classical monocytes display inflammatory features: Validation in Sepsis and Systemic Lupus Erythematous. Scientific reports. 2015;5:13886 Epub 2015/09/12. doi: 10.1038/srep13886 . 26358827 10.1038/srep13886 PMC4566081

[19] van Lith M, McEwen-Smith RM, Benham AM. HLA-DP, HLA-DQ, and HLA-DR have different requirements for invariant chain and HLA-DM. The Journal of biological chemistry. 2010;285(52):40800–8. Epub 2010/10/21. doi: 10.1074/jbc.M110.148155 . 20959457 10.1074/jbc.M110.148155 PMC3003381

[20] Gren ST, Rasmussen TB, Janciauskiene S, Hakansson K, Gerwien JG, Grip O. A Single-Cell Gene-Expression Profile Reveals Inter-Cellular Heterogeneity within Human Monocyte Subsets. PloS one. 2015;10(12):e0144351 Epub 2015/12/10. doi: 10.1371/journal.pone.0144351 . 26650546 10.1371/journal.pone.0144351 PMC4674153

[21] Liu B, Dhanda A, Hirani S, Williams EL, Sen HN, Martinez Estrada F, et al CD14++CD16+ Monocytes Are Enriched by Glucocorticoid Treatment and Are Functionally Attenuated in Driving Effector T Cell Responses. J Immunol. 2015;194(11):5150–60. Epub 2015/04/26. doi: 10.4049/jimmunol.1402409 . 25911752 10.4049/jimmunol.1402409 PMC4433824

[22] Abeles RD, McPhail MJ, Sowter D, Antoniades CG, Vergis N, Vijay GK, et al CD14, CD16 and HLA-DR reliably identifies human monocytes and their subsets in the context of pathologically reduced HLA-DR expression by CD14(hi) /CD16(neg) monocytes: Expansion of CD14(hi) /CD16(pos) and contraction of CD14(lo) /CD16(pos) monocytes in acute liver failure. Cytometry Part A: the journal of the International Society for Analytical Cytology. 2012;81(10):823–34. Epub 2012/07/28. doi: 10.1002/cyto.a.22104 . 22837127 10.1002/cyto.a.22104

[23] Tolouei Semnani R, Moore V, Bennuru S, McDonald-Fleming R, Ganesan S, Cotton R, et al Human monocyte subsets at homeostasis and their perturbation in numbers and function in filarial infection. Infection and immunity. 2014;82(11):4438–46. Epub 2014/08/13. doi: 10.1128/IAI.01973-14 . 25114121 10.1128/IAI.01973-14 PMC4249311

[24] Surh CD, Sprent J. Homeostatic T Cell Proliferation: How Far Can T Cells Be Activated to Self-Ligands? The Journal of Experimental Medicine. 2000;192(4):9–14. 10.1084/jem.192.4.f9 PMC2193242 10952731

[25] Reading JL, Vaes B, Hull C, Sabbah S, Hayday T, Wang NS, et al Suppression of IL-7-dependent Effector T-cell Expansion by Multipotent Adult Progenitor Cells and PGE2. Mol Ther. 2015;23(11):1783–93. doi: 10.1038/mt.2015.131 26216515 10.1038/mt.2015.131 PMC4817941

[26] Thomas G, Tacke R, Hedrick CC, Hanna RN. Nonclassical Patrolling Monocyte Function in the Vasculature. Arteriosclerosis, thrombosis, and vascular biology. 2015;35(6):1306–16. doi: 10.1161/ATVBAHA.114.304650 25838429 10.1161/ATVBAHA.114.304650 PMC4441550

[27] Hershman MJ, Appel SH, Wellhausen SR, Sonnenfeld G, Polk HC Jr. Interferon-gamma treatment increases HLA-DR expression on monocytes in severely injured patients. Clinical and experimental immunology. 1989;77(1):67–70. Epub 1989/07/01. . 2504520 PMC1541937

[28] Martinez FO, Gordon S. The M1 and M2 paradigm of macrophage activation: time for reassessment. F1000prime reports. 2014;6:13 Epub 2014/03/29. doi: 10.12703/P6-13 . 24669294 10.12703/P6-13 PMC3944738

[29] Murray PJ, Allen JE, Biswas SK, Fisher EA, Gilroy DW, Goerdt S, et al Macrophage activation and polarization: nomenclature and experimental guidelines. Immunity. 2014;41(1):14–20. Epub 2014/07/19. doi: 10.1016/j.immuni.2014.06.008 . 25035950 10.1016/j.immuni.2014.06.008 PMC4123412

[30] Cao ZA, Moore BB, Quezada D, Chang CH, Jones PP. Identification of an IFN-gamma responsive region in an intron of the invariant chain gene. European journal of immunology. 2000;30(9):2604–11. Epub 2000/09/29. doi: 10.1002/1521-4141(200009)30:9<2604::AID-IMMU2604>3.0.CO;2-6 . 11009094 10.1002/1521-4141(200009)30:9<2604::AID-IMMU2604>3.0.CO;2-6

[31] Lah TT, Hawley M, Rock KL, Goldberg AL. Gamma-interferon causes a selective induction of the lysosomal proteases, cathepsins B and L, in macrophages. FEBS letters. 1995;363(1–2):85–9. Epub 1995/04/17. . 7729559 10.1016/0014-5793(95)00287-j

[32] Wicks IP, Roberts AW. Targeting GM-CSF in inflammatory diseases. Nature reviews Rheumatology. 2016;12(1):37–48. Epub 2015/12/04. doi: 10.1038/nrrheum.2015.161 . 26633290 10.1038/nrrheum.2015.161

[33] Meisel C, Schefold JC, Pschowski R, Baumann T, Hetzger K, Gregor J, et al Granulocyte-macrophage colony-stimulating factor to reverse sepsis-associated immunosuppression: a double-blind, randomized, placebo-controlled multicenter trial. American journal of respiratory and critical care medicine. 2009;180(7):640–8. Epub 2009/07/11. doi: 10.1164/rccm.200903-0363OC . 19590022 10.1164/rccm.200903-0363OC

[34] Spies C, Luetz A, Lachmann G, Renius M, von Haefen C, Wernecke KD, et al Influence of Granulocyte-Macrophage Colony-Stimulating Factor or Influenza Vaccination on HLA-DR, Infection and Delirium Days in Immunosuppressed Surgical Patients: Double Blind, Randomised Controlled Trial. PloS one. 2015;10(12):e0144003 Epub 2015/12/08. doi: 10.1371/journal.pone.0144003 . 26641243 10.1371/journal.pone.0144003 PMC4671639

[35] Lachmann G, Kurth J, von Haefen C, Yuerek F, Wernecke KD, Spies C. In vivo application of Granulocyte-Macrophage Colony-stimulating Factor enhances postoperative qualitative monocytic function. International journal of medical sciences. 2017;14(4):367–75. Epub 2017/05/30. doi: 10.7150/ijms.18288 . 28553169 10.7150/ijms.18288 PMC5436479

[36] Sánchez-Torres C, García-Romo GS, Cornejo-Cortés MA, Rivas-Carvalho A, Sánchez-Schmitz G. CD16+ and CD16− human blood monocyte subsets differentiate in vitro to dendritic cells with different abilities to stimulate CD4+ T cells. International Immunology. 2001;13(12):1571–81. doi: 10.1093/intimm/13.12.1571 11717198 10.1093/intimm/13.12.1571

[37] Fromm SV, Ehrlich R. IFN-gamma affects both the stability and the intracellular transport of class I MHC complexes. Journal of interferon & cytokine research: the official journal of the International Society for Interferon and Cytokine Research. 2001;21(4):199–208. Epub 2001/05/22. doi: 10.1089/107999001750169790 . 11359650 10.1089/107999001750169790

[38] Muczynski KA, Anderson SK, Pious D. Discoordinate surface expression of IFN-gamma-induced HLA class II proteins in nonprofessional antigen-presenting cells with absence of DM and class II colocalization. J Immunol. 1998;160(7):3207–16. Epub 1998/04/08. . 9531276

[39] Koppelman B, Neefjes JJ, de Vries JE, de Waal Malefyt R. Interleukin-10 Down-Regulates MHC Class II αβ Peptide Complexes at the Plasma Membrane of Monocytes by Affecting Arrival and Recycling. Immunity. 1997;7(6):861–71. http://dx.doi.org/10.1016/S1074-7613(00)80404-5. 9430231 10.1016/s1074-7613(00)80404-5

[40] Sprangers S, de Vries TJ, Everts V. Monocyte Heterogeneity: Consequences for Monocyte-Derived Immune Cells. Journal of immunology research. 2016;2016:1475435 Epub 2016/08/02. doi: 10.1155/2016/1475435 . 27478854 10.1155/2016/1475435 PMC4958468

[41] de Waal Malefyt R, Haanen J, Spits H, Roncarolo MG, te Velde A, Figdor C, et al Interleukin 10 (IL-10) and viral IL-10 strongly reduce antigen-specific human T cell proliferation by diminishing the antigen-presenting capacity of monocytes via downregulation of class II major histocompatibility complex expression. J Exp Med. 1991;174(4):915–24. Epub 1991/10/01. . 1655948 10.1084/jem.174.4.915 PMC2118975

[42] Spencer JV, Lockridge KM, Barry PA, Lin G, Tsang M, Penfold ME, et al Potent immunosuppressive activities of cytomegalovirus-encoded interleukin-10. Journal of virology. 2002;76(3):1285–92. Epub 2002/01/05. doi: 10.1128/JVI.76.3.1285-1292.2002 . 11773404 10.1128/JVI.76.3.1285-1292.2002 PMC135865

[43] Stansfield BK, Ingram DA. Clinical significance of monocyte heterogeneity. Clinical and translational medicine. 2015;4:5 Epub 2015/04/09. doi: 10.1186/s40169-014-0040-3 . 25852821 10.1186/s40169-014-0040-3 PMC4384980

[44] Thomas GD, Hamers AAJ, Nakao C, Marcovecchio P, Taylor AM, McSkimming C, et al Human Blood Monocyte Subsets: A New Gating Strategy Defined Using Cell Surface Markers Identified by Mass Cytometry. Arterioscler Thromb Vasc Biol. 2017 Epub 2017/06/10. doi: 10.1161/atvbaha.117.309145 . 28596372 10.1161/ATVBAHA.117.309145 PMC5828170

