# Supplementary figures and images for: The MHC class II antigen presentation pathway in human monocytes differs by subset and is regulated by cytokines
Source: PLoS One. 2017 Aug 23;12(8):e0183594. doi: 10.1371/journal.pone.0183594 (PMC5568224; doi:10.1371/journal.pone.0183594)

S1 Fig – Lee et al

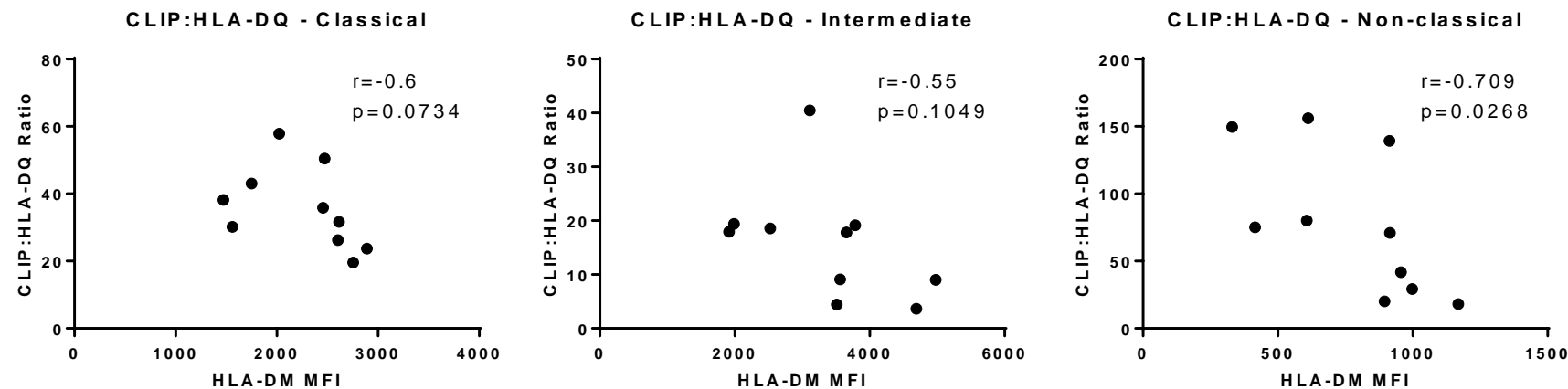

Supplement: S1 Fig — Spearman correlation between the CLIP/DQ ratios (multiplied by 100) and HLA-DM MFI’s for the three monocyte subsets from 10 samples. (PDF) [file pone.0183594.s001.pdf]

S2 Fig – Lee et al

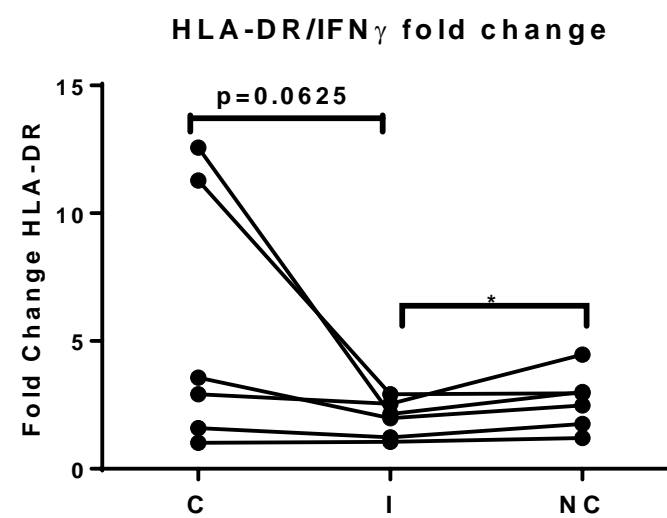

Supplement: S2 Fig — Sorted monocytes from each subset were incubated with 5 ng/ml of IFNγ for 20–24 hours or left unstimulated. Cells were stained for surface HLA-DR using L243, and median fluorescence intensity (MFI) was obtained. Fold change for HLA-DR was obtained by dividing the HLA-DR MFI after IFNγ stimulation by the MFI of the unstimulated subset. Six independent samples were tested. Wilcoxon matched-pairs signed rank test was used for group to group comparison. Statistical significance represented by asterisk: *, p < 0.05. C: Classical; I: Intermediate; NC: Non-classical. (PDF) [file pone.0183594.s002.pdf]

S3 Fig – Lee et al

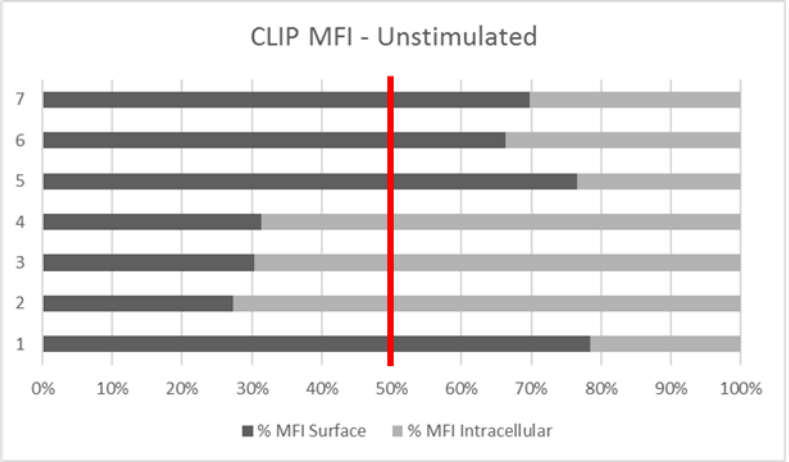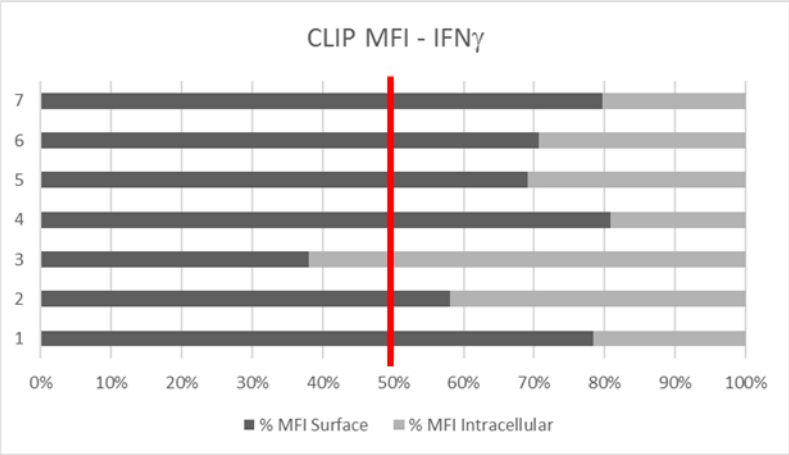

Supplement: S3 Fig — (A) CLIP expression was determined using CerCLIP antibody in unstimulated, positively isolated CD14+ monocytes, both at the surface and intracellularly (defined as the difference between total CLIP MFI and the surface MFI) after 20–24 hours; MFI expressed as percentage. (B) CD14+ monocytes were stimulated with 5 ng/ml of IFNγ for 20–24 hours and CLIP expression determined as in (A). (PDF) [file pone.0183594.s003.pdf]
